# Relationship between Preoperative Red Cell Distribution Width and Prolonged Postoperative Use of Catecholamines in Minimally Invasive Mitral Valve Surgery Patients: A Retrospective Cohort Study

**DOI:** 10.3390/jcm13195736

**Published:** 2024-09-26

**Authors:** Alfonso Carrara, Lorenzo Peluso, Federica Baccanelli, Matteo Parrinello, Giuseppe Santarpino, Laura Giroletti, Ascanio Graniero, Alfonso Agnino, Giovanni Albano

**Affiliations:** 1 Department of Anesthesia and Intensive Care, Humanitas Gavazzeni, Via M. Gavazzeni, 21, 24125 Bergamo, Italylorenzopeluso80@gmail.com (L.P.); matteo.parrinello@gavazzeni.it (M.P.); giovanni.albano@gavazzeni.it (G.A.); 2 Department of Biomedical Sciences, Humanitas University, Via Rita Levi Montalcini, 4, Pieve Emanuele, 20072 Milan, Italy; 3 Department of Experimental and Clinical Medicine, Magna Graecia University, Viale Europa, 88100 Catanzaro, Italy; 4 Department of Cardiac Surgery, Paracelsus Medical University, 90419 Nuremberg, Germany; 5 Department of Cardiac Surgery, Humanitas Gavazzeni, Via M. Gavazzeni, 21, 24125 Bergamo, Italy; laura.giroletti@gavazzeni.it (L.G.); ascanio.graniero@gavazzeni.it (A.G.); alfonso.agnino@gavazzeni.it (A.A.)

**Keywords:** RDW, cardiac surgery, robotic surgery

## Abstract

**Background/Objectives**: Elevated RDW has emerged in cardiac surgery as a potential means of preoperative risk stratification with the capacity to predict short- and long-term postoperative mortality, acute kidney injury, and postoperative atrial fibrillation. The question as to whether perioperative hemodynamic instability may be predicted by such a marker remains a topic of ongoing debate. The aim of this study was to explore the relationship between preoperative RDW and prolonged postoperative catecholamine use in minimally invasive mitral valve surgery. **Methods**: We performed a retrospective monocentric cohort study in an academic hospital; we enrolled patients who had undergone minimally invasive mitral valve surgery (including both robot-assisted and non-robot-assisted procedures) between January 2019 and December 2022. We considered the use of inotropes and/or vasopressors for at least twelve hours after post-surgery ICU admission to qualify as the prolonged postoperative use of catecholamines (PPUC). The RDW was obtained from the routine full blood count analysis performed upon admission or a maximum of 72 h before surgery. We also performed a multivariable logistic regression analysis with PPUC as the dependent variable. **Results**: We finally enrolled 343 patients. Upon multivariate analysis, RDW >14.4% was independently associated with prolonged postoperative catecholamine use when compared to the reference group (OR 2.62 [1.06–4.84]; *p* = 0.03). Moreover, the EuroSCORE II score (OR 1.38 [1.03–1.85]; *p* = 0.03), the cross-clamp time (OR 1.01 [1.01–1.02]; *p* < 0.01), and robot-assisted mitral valve surgery (OR 0.53 [0.30–0.93]; *p* < 0.03) were independently associated with the prolonged postoperative use of catecholamines. **Conclusions**: This study identified that an elevated preoperative RDW (>14.4%), the EuroSCORE II score, and the cross-clamp time independently predict prolonged postoperative catecholamine use in minimally invasive mitral valve surgery patients. Conversely, the robot-assisted approach was associated with a smaller hemodynamic impairment.

## 1. Introduction

Cardiac surgery, which is performed on 1 to 1.25 million patients worldwide every year [1], faces a major challenge due to the global aging population, which is resulting in higher-risk cases with more comorbidities. Elderly patients exhibit reduced physiologic reserves, elevating the risk of complications and mortality. The repercussions extend to healthcare costs, because, as the population undergoing cardiac surgery ages, they become more prone to postoperative complications. Hemodynamic instability in cardiac surgery, requiring catecholamines, represents a formidable complication that substantially escalates mortality, especially when associated with separation from cardiopulmonary bypass (CPB) [2,3]. Recognizing, predicting, understanding, preventing, and treating such instability is pivotal in enhancing patient selection and care, particularly for this high-risk demographic.

In cardiovascular intensive care units (CICUs), where one in four patients receives vasopressors or inotropes, the greater requirements of patients are linked to elevated mortality, emphasizing the significance of optimal circulatory support therapy. During cardiac surgery, over 50% of patients receive inotropes, primarily for postoperative low cardiac output syndrome (LCOS) [4]. However, the choice and duration of inotropic therapy lack definitive evidence, highlighting the need for high-quality studies in this domain [5,6]. Specifically, in mitral valve surgery, the use of vasopressors and inotropes is often required [7]. Finally, the duration of support is usually short, and very few studies have assessed the risk factors for the need for prolonged vasoactive support [8,9].

In this context, identifying new, clinically practical, and cost-effective markers may be helpful in predicting postoperative instability. The red cell distribution width (RDW), a biomarker reflecting erythropoietic dysfunction, has emerged as a promising parameter. Elevated RDW, historically used for the diagnosis of anemia, has attracted attention for its predictive potential in various medical disciplines [10,11,12]. Studies have linked an elevated RDW to adverse outcomes after cardiac valve surgery, and it has various associations with cardiovascular risks across different patient populations [13,14]. In evaluating inflammatory activity more broadly, alongside its prognostic implications for ischemic disorders and cerebrovascular disease [15], elevated RDW has emerged as a potential means of preoperative risk stratification and predicting short- and long-term postoperative mortality [16], acute kidney injury [17], and postoperative atrial fibrillation [18]. While its precise mechanisms are not yet fully understood, the RDW is increasingly recognized as a global marker of oxidative stress and chronic inflammation [4]. In this context, the question as to whether perioperative hemodynamic instability, which necessitates the use of catecholamines, is associated with preoperative oxidative stress or underdiagnosed chronic inflammation remains a topic of ongoing debate.

Therefore, the aim of this study was to explore the relationship between the preoperative RDW and prolonged postoperative catecholamine use in minimally invasive mitral valve surgery.

## 2. Methods

### 2.1. Study Design and Patient Selection

**Figure 1:**
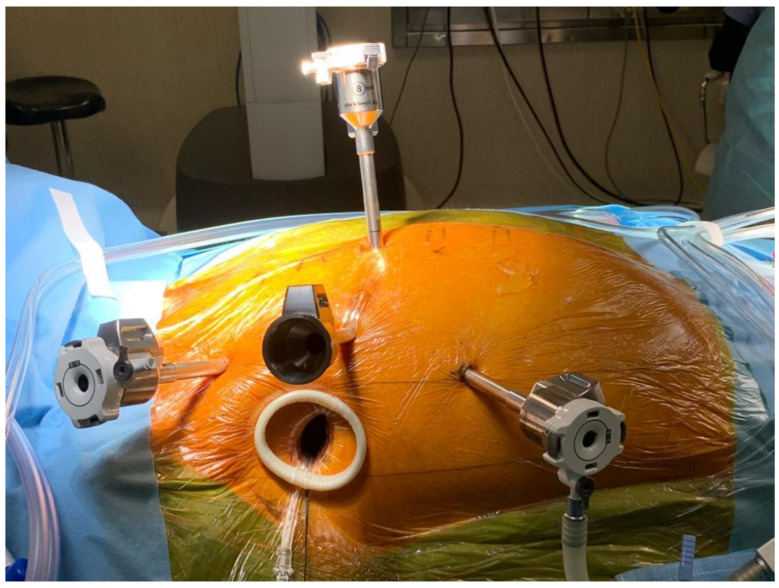
Robot-assisted procedure setting.

**Figure 2:**
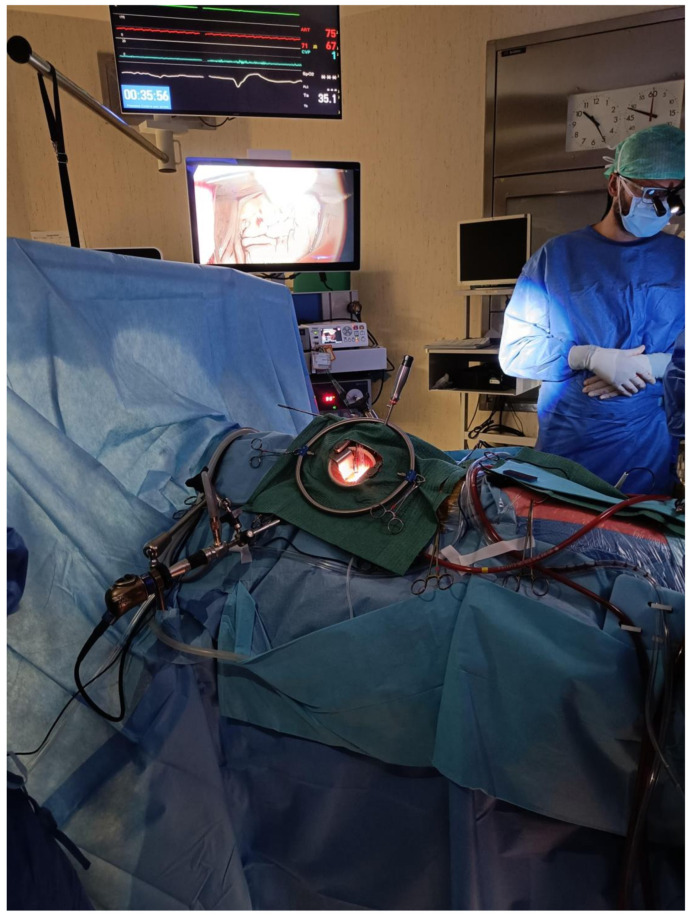
Non-robot-assisted procedure setting.

This retrospective cohort study was performed in an academic hospital. From our institutional database (January 2019 to December 2022), all adult (>18 years old) patients with moderate to severe mitral valve regurgitation detected during a transesophageal echocardiography examination and undergoing minimally invasive mitral valve surgery (including both robot-assisted and non-robot-assisted procedures, as shown in Figure 1 and Figure 2) were considered eligible for this study.

The exclusion criteria were the absence of preoperative RDW measurements in the 72 h before surgery, the need for erythrocyte transfusion in the three months prior to surgery, and patients who had undergone surgery in an emergency or urgent setting.

### 2.2. Patient Care and Monitoring

Patients scheduled for elective procedures were admitted to the hospital on the day before the surgery. Preoperative assessment for all patients undergoing cardiac surgery included blood laboratory tests, electrocardiography, transthoracic echocardiography, transesophageal echocardiography, carotid duplex ultrasound, thoracic and abdomen CT scans, and coronary angiography.

Intraoperatively, all patients were monitored with invasive arterial blood pressure through a radial artery catheter placed prior to the administration of general anesthesia. Moreover, a central venous catheter was placed in the right internal jugular vein for central venous pressure (CVP) monitoring and for the provision of drugs; the body temperature was monitored using a bladder catheter. The assessment of cardiac function was performed via repeated transesophageal echocardiography, while cerebral function monitoring was performed with quantitative electroencephalography (qEEG) and subsequent near-infrared spectroscopy (NIRS).

General anesthesia was induced using a combination of sedatives (midazolam and propofol) and analgesics (fentanyl) and maintained with a continuous infusion of propofol and fentanyl boluses, which were adjusted according to the qEEG and clinical response. After surgery, patients were transferred to the intensive care unit. Here, after rewarming and hemodynamic optimization guided by cardiac ultrasound and analgesic therapy, patients were weaned off mechanical ventilation and extubated within 6 h. Mechanical ventilation was maintained in patients with severe hemodynamic impairment until resolution, as decided by clinical judgement. The majority of patients were discharged within 48 h of ICU admission. Further information on the surgical techniques employed can be found elsewhere [19,20,21].

### 2.3. Data Collection and Definition

We collected data on demographics, comorbidities, laboratory tests, the duration of CPB, the aortic cross-clamp and operative time, blood loss, the use of red blood cell transfusions (RBCTs), the development of acute kidney injury (AKI), the use of catecholamines, the ICU and hospital length of stay, and hospital mortality. Among the collected variables, RDW values, along with other relevant clinical and laboratory information, were extracted from electronic patient records and the healthcare information system. 

We defined “major bleeding” as blood loss of more than 500 mL in the first 24 h of ICU admission and/or the need for surgical revision during the ICU stay. 

To reduce the bias of postoperative hypovolemia and the impact of postoperative sedatives on the hemodynamics, we considered the use of inotropes and/or vasopressors for at least twelve hours after post-surgery ICU admission to be prolonged postoperative use of catecholamines (PPUC). Other patients were placed in the “no prolonged postoperative use of catecholamines” (NPPUC) group. 

The RDW was obtained from the routine full blood count analysis taken upon admission or a maximum of 72 h before surgery. Complete blood counts were performed using the UniCel DXH 600 Coulter Cellular Analysis System (Beckman Coulter International S.A., Nyon, France). RDW values were calculated using the following formula: RDW = (standard deviation of RBC corpuscular volume)/(mean corpuscular volume [MCV]) × 100 (%). The normal range of RDW values in our laboratory was 11–16.5%. 

### 2.4. Endpoints of the Study

The primary endpoint of the study was to assess whether the preoperative RDW was associated with the prolonged postoperative use of catecholamines in minimally invasive mitral valve surgery patients. The secondary endpoint was a subgroup analysis used to assess whether the preoperative RDW was associated with the prolonged postoperative use of catecholamines in patients without “major bleeding”.

### 2.5. Statistical Analysis

Discrete variables are expressed as a count (percentage) and continuous variables as the mean  ±  standard deviation (SD) or median (25th–75th percentiles). The Kolmogorov–Smirnov test was used, and histograms and normal quantile plots were examined to verify the normality of the distribution of continuous variables. Differences in demographics and clinical and RDW patterns between groups (i.e., PPUC vs. NPPUC) were assessed using the chi-square test, Fisher’s exact test, Student’s t-test, or the Mann–Whitney U-test, as appropriate. Moreover, we divided the RDW values into quartiles and considered their distribution in the two different groups.

We performed multivariable logistic regression analysis with PPUC as the dependent variable; collinearity between variables (i.e., a linear correlation coefficient higher than 0.3) was excluded prior to modeling. Only variables associated with PPUC upon univariate analysis (*p*  <  0.05) were included in the multivariate model. Odds ratios (OR) with 95% confidence intervals (CI) were computed using an enter model. A similar approach was used to perform a multivariate analysis, with PPUC as the dependent variable within the subgroup analysis. We tested the fitness of the model using the Hosmer and Lemeshow goodness-of-fit test. The ORs for the RDW categories were calculated using the first quartile as a reference. All statistical tests were two-tailed, and a *p*-value of  <0.05 was considered statistically significant. Data were analyzed using IBM SPSS Statistics for Macintosh 25 (Armonk, NY, USA) and GraphPad PRISM version 8.0 (San Diego, CA, USA).

## 3. Results

Of the 349 patients who met our inclusion criteria, six were excluded because their RDW values were unavailable (Supplementary Figure S1). Finally, a total of 343 patients were included in the analysis; the median age was 63 [53–71] years, and 129 (38%) were female.

The preoperative characteristics of the study population are shown in Table 1; 76 (22%) patients had atrial fibrillation, and 197 (57%) presented with arterial hypertension. The median preoperative RDW was 13.7 [13.2–14.4]%. Overall, 87 (25%) patients had an RDW between 13.8 and 14.4%, and 90 (26%) patients had an RDW > 14.4. The median EuroSCORE II score was 0.89 [0.64–1.32], and the median left ventricular ejection fraction was 62 [60–66]%.

A total of 129 patients (38%) underwent robot-assisted mitral valve surgery, whereas 18 (5%) patients underwent mitral valve replacement. The median duration of cardiopulmonary bypass was 140 [119–169] minutes; the same measure for the cross-clamp time was 88 [77–104] minutes. In our study, 102 (30%) patients developed hemodynamic instability requiring the prolonged postoperative use of catecholamines.

### 3.1. Prolonged Postoperative Use of Catecholamines vs. No Prolonged Postoperative Use of Catecholamines

The patients in the PPUC group were older than the patients in the NPPUC group (64 [56–74] vs. 62 [51–70]; *p* < 0.01). Regarding comorbidities, atrial fibrillation was more common in the PPUC group, [30 (29%) vs. 46 (19%); *p* = 0.04], and arterial hypertension was more common in the PPUC group, [69 (68%) vs. 128 (53%); *p* = 0.02]. The PPUC group showed also a significantly higher EuroSCORE II score (1.10 [0.70–1.82] vs. 0.81 [0.60–1.12]; *p* < 0.01), whereas the left ventricular ejection fraction was lower (60 [57–65] vs. 63 [60–67]; *p* = 0.02)%.

**Figure 3:**
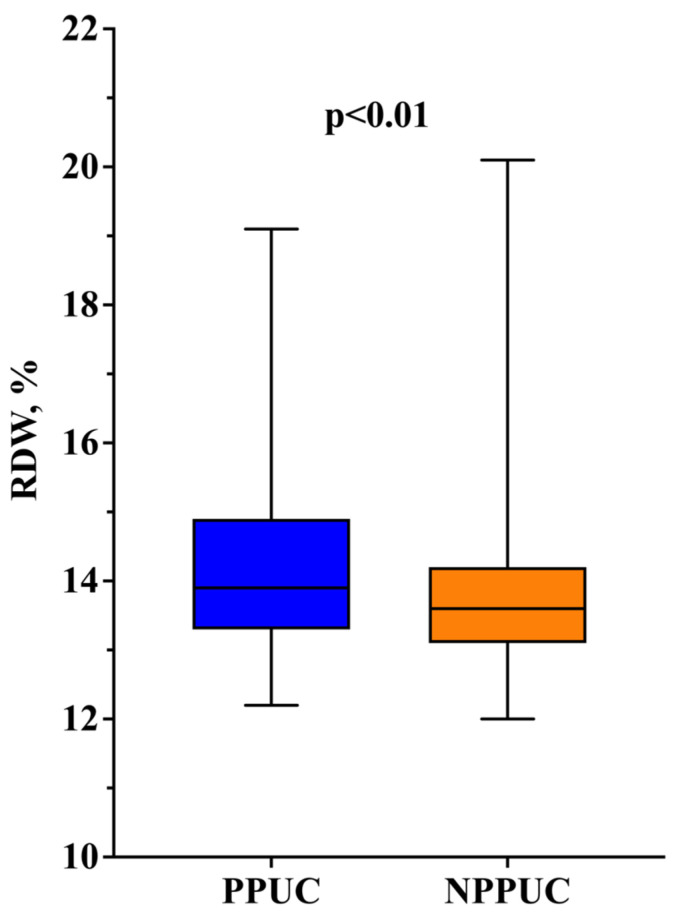
Differences in preoperative RDW in patients with prolonged postoperative use of catecholamines (PPUC) vs. patients without prolonged postoperative use of catecholamines (NPPUC).

**Figure 4:**
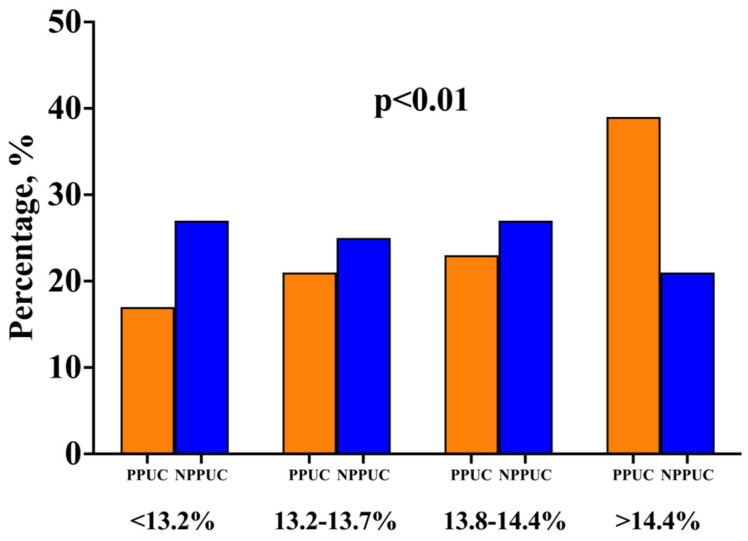
Quartile distribution of preoperative RDW in patients with prolonged postoperative use of catecholamines (PPUC) vs. patients without prolonged postoperative use of catecholamines (NPPUC).

The preoperative RDW values were significantly higher in the PPUC group (13.9 [13.3–14.9] vs. 13.6 [13.1–14.2]; *p* < 0.01)% (Figure 3). When dividing patients based on the median preoperative RDW value, we observed that those with RDW levels greater than 14.4 were more frequently administered prolonged postoperative catecholamines (36% vs. 19%; *p* < 0.01). Moreover, upon dividing the RDW values into quartiles of distribution, we found that the PPUC group presented a larger proportion of patients with a high preoperative RDW (as shown in Table 1 and Figure 4). Regarding the intraoperative characteristics, PPUC patients underwent fewer robot-assisted mitral valve surgery procedures than NPPUC patients [27 (27%) vs. 102 (42%); *p* < 0.01].

Conversely, the PPUC group underwent more mitral valve replacement procedures [10 (10%) vs. 8 (3%), *p* = 0.03]. The PPUC group underwent significantly longer cardiopulmonary bypass procedures (152 [120–178] vs. 137 [118–165] minutes, *p* = 0.03) and cross-clamping (95 [78–115] vs. 86 [77–100] minutes; *p* < 0.01).

Upon multivariate analysis, we found an RDW >14.4% to be independently associated with PPUC when compared with the reference group (OR 2.62 [1.06–4.84]; *p* = 0.03) (Table 2). Moreover, the EuroSCORE II score (OR 1.38 [1.03–1.85]; *p* = 0.03), cross-clamp time (OR 1.01 [1.01–1.02]; *p* < 0.01), and robot-assisted mitral valve surgery (OR 0.53 [0.30 –0.93]; *p* = 0.03) were independently associated with the prolonged postoperative use of catecholamines.

### 3.2. Postoperative Outcomes

Patients in the PPUC group had higher lactate levels within 24 h (4.0 [2.4–6.3] vs. 2.6 [1.9–3.8] mmol/L; *p* < 0.01–Supplementary Table S1), and they developed AKI more frequently [16 (16%) vs. 6 (3%); *p* < 0.01]. Moreover, patients with PPUC were mechanically ventilated longer than those in the NPPUC group (8 [4–13] vs. 4 [3–6] hours; *p* < 0.01). They had longer ICU stays (47 [44–72] vs. 43 [34–46] hours; *p* < 0.01) and longer postoperative in-hospital stays (9 [8–12] vs. 8 [7–10] days; *p* < 0.01). Finally, PPUC patients were less frequently discharged directly to their homes [52 (51%) vs. 169 (70); *p* < 0.01], instead more frequently requiring rehabilitation in specific institutions. The catecholamine dosages and timings are shown in Table S2 of the Supplementary Materials.

### 3.3. Subgroup Analysis

Considering patients without major bleeding, 60 (24%) patients required the prolonged postoperative use of catecholamines. Atrial fibrillation [22 (37%) vs. 37 (20%); *p* < 0.01] and arterial hypertension [43 (72%) vs. 96 (51%); *p* < 0.01] were significantly more common in the PPUC group.

The PPUC group had a significantly higher EuroSCORE II score (0.97 [0.70–1.57] vs. 0.82 [0.60–1.11]; *p* = 0.02). The preoperative RDW was also higher in the PPUC group (13.9 [13.5–14.8] vs. 13.6 [13.1–14.1], *p* = 0.01). Observing the specific quartiles within which the preoperative RDW values were distributed, the PPUC group had a larger proportion of patients with an elevated preoperative RDW.

PPUC patients underwent fewer robot-assisted mitral valve surgery procedures [12 (20%) vs. 75 (40%); *p* < 0.01], and they underwent longer cardiopulmonary bypass (144 [123–175] vs. 133 [113–159] minutes; *p* = 0.03) and cross-clamp (97 [80–114] vs. 85 [75–98] minutes; *p* < 0.01) procedures when compared with NPPUC patients (see Table S3 in the Supplementary Materials).

Upon multivariate analysis, we found that the length of cross-clamp procedures (OR 1.02 [1.01–1.03], *p* < 0.01) and robot-assisted mitral valve surgery (OR 0.45 [0.21–0.97], *p* = 0.04), but not the RDW values, had a significant association with postoperative hemodynamic instability that required the prolonged postoperative use of catecholamines (see Supplementary Table S4).

## 4. Discussion

In our study, we showed that RDW values of more than 14.4% had a significant association with the prolonged postoperative use of catecholamines in minimal invasive mitral valve surgery, when compared with the baseline. Moreover, we showed that the EuroSCORE II score and cross-clamp duration were independently associated with the prolonged postoperative use of catecholamines, whereas robot-assisted mitral valve surgery was associated with less instability requiring catecholamines. Through subgroup analysis, these results were partially confirmed; in fact, the cross-clamp duration, but not RDW, was significantly associated with the prolonged postoperative use of catecholamines. On the other hand, robot-assisted mitral valve surgery was independently associated with the lower postoperative use of catecholamines.

Recently, there has been renewed interest in the RDW as an inflammatory marker. The RDW is now a known marker of autoimmune or inflammatory conditions such as systemic lupus erythematosus and rheumatoid arthritis [22]. Indeed, inflammation itself causes the impairment of erythropoiesis, which is likely mediated by proinflammatory cytokines such as IL-1, IL-6, TNF-a, and IFN-c [23,24]. Moreover, the RDW has been strongly correlated with prognoses in cardiovascular disease and ischemic stroke, which both have a strong inflammatory and thromboembolic component [25,26].

Historically, increased anisocytosis has been associated with several conditions, such as deficiencies in iron, vitamin B12, or folate [27]; hemolysis; and prior blood transfusions. It is usually caused by the chronic impairment of erythropoiesis [28,29]; this could imply that the RDW is suggestive of a chronic rather than an acute process. Nevertheless, higher RDW values were also correlated with higher acute-phase inflammatory markers, such as high-sensitivity C-reactive protein (hsCRP) and the erythrocyte sedimentation rate (ESR), and did so independently of confounding factors [30]. In this context, the RDW may reflect a proinflammatory state and oxidative stress caused by an intermittent, undiagnosed condition [31].

In cardiac surgery patients, several studies have shown that a preoperative elevated RDW is associated with different postoperative outcomes [16], particularly long-term and short-term mortality [32], acute kidney injury [33], and postoperative atrial fibrillation [18].

Currently, there is no consensus on the definition of the prolonged postoperative use of catecholamines; indeed, other studies have considered a minimum of 24 h to qualify [9]. In our investigation, we opted to consider a 12 h interval, as it represented the time limit following circulatory volume optimization and the time after patients’ warming and weaning process from mechanical ventilation.

Interestingly, our findings were partially validated by a subgroup analysis of patients without major bleeding; the RDW, in fact, exhibited a difference between the two groups but did not show an independent association when adjusted for confounding factors. The mechanisms underlying our results are speculative at best; we hypothesized that patients experiencing bleeding events were potentially more prone to heightened inflammation.

By excluding this subgroup from our analysis, it is likely that we eliminated individuals with elevated inflammation and, consequently, higher RDW levels. Another plausible explanation for this result may be our reduced sample size and its limited statistical power.

The mechanisms elucidating the connection between elevated RDW levels and prolonged postoperative catecholamine use remain incompletely understood. Furthermore, whether the RDW serves only as a marker for frailty and underlying inflammation or if it actively contributes to morbidity and mortality, thereby impairing microcirculation, remains the subject of debate [34].

Considering other risk factors, the duration of aortic cross-clamping is a well-established cause of inflammatory responses previously described as a cause of postoperative hemodynamic impairment [35]. To our knowledge, this is the first study to report an association between robot-assisted mitral valve surgery and the reduced use of postoperative catecholamines.

This study has several limitations. Firstly, its retrospective design may have resulted in overlooking variables that could influence prolonged postoperative catecholamine use. Secondly, as it was a monocentric study, its generalizability to other centers may be limited. Thirdly, this study primarily focused on patients undergoing minimally invasive mitral valve surgery, which constrains the applicability of our results; however, it does provide insights into a specific subgroup of the cardiac surgery population. Fourth, a notable challenge in utilizing the RDW as a biomarker is the absence of a universal reference range due to a lack of consensus between manufacturers and laboratories. Fifth, while our study specifically investigated RDW values, integrating these values with other well-established predictors of postoperative hemodynamic impairment may improve the accuracy of our predictions. Sixth, we did not specifically assess trends in the RDW values in the initial days after ICU admission; such changes, although potentially confounded by transfusions, could yield valuable information. Seventh, we did not consider the potential impact of certain medications on preoperative RDW levels. Finally, we did not have access to data on the preoperative levels of ferritin and other parameters that are potentially related to anisocytosis.

## 5. Conclusions

In our study, we demonstrated a significant association between RDW levels exceeding 14.4% and prolonged postoperative catecholamine use in minimally invasive mitral valve surgery compared to baseline values. Additionally, our findings indicated that the EuroSCORE II score and cross-clamp time are independently associated with the extended postoperative use of catecholamines, while robot-assisted mitral valve surgery is linked to reduced hemodynamic impairment. Notably, the predictive capacity of the RDW was not validated in patients without major bleeding. Future prospective studies are crucial if we are to further elucidate the role of the RDW and explore its potential applications in the context of minimally invasive mitral valve surgery.

## Figures and Tables

**Table 1:** Preoperative and intraoperative characteristics of the study population, according to the development of postoperative hemodynamic instability.

	OVERALL (n = 343)	PPUC (n = 102)	NPPUC (n = 241)	*p*-Value
Age, years	63 [53–71]	64 [56–74]	62 [51–70]	<0.01
Female gender, n (%)	129 (38)	42 (41)	87 (36)	0.40
BMI	24.4 [22.1–26.6]	24.0 [21.9–26.3]	24.6 [22.4–26.8]	0.32
Preoperative Characteristics				
Atrial fibrillation, n (%)	76 (22)	30 (29)	46 (19)	0.04
Mitral valve stenosis, n (%)	12 (4)	6 (6)	6 (3)	0.19
Arterial hypertension, n (%)	197 (57)	69 (68)	128 (53)	0.02
Chronic arteriopathy, n (%)	4 (1)	1 (1)	3 (1)	1.00
Diabetes, n (%)	18 (5)	4 (4)	14 (6)	0.60
Neurologic disease, n (%)	29 (9)	9 (9)	20 8)	0.84
Asthma/COPD, n (%)	28 (8)	11 (11)	17 (7)	0.28
Active smoker, n (%)	48 (14)	16 (16)	32 (13)	0.61
Preoperative hemoglobin	14.0 [13.1–15.0]	14.0 [13.0–14.9]	14.1 [13.1–15.0]	0.33
Preoperative creatinine	0.90 [0.80–1.06]	0.92 [0.80–1.15]	0.89 [0.80–1.03]	0.09
Preoperative RDW	13.7 [13.2–14.4]	13.9 [13.3–14.9]	13.6 [13.1–14.2]	<0.01
Preoperative RDW				<0.01
<13.2%	83 (24)	17 (17)	66 (27)	
13.2–13.7%	83 (24)	22 (21)	61 (25)	
13.8–14.4%	87 (25)	23 (23)	64 (27)	
>14.4%	90 (26)	40 (39)	50 (21)	
Preoperative CRP	0.14 [0.06–0.29]	0.17 [0.07–0.32]	0.12 [0.06–0.26]	0.21
EuroSCORE II, score	0.89 [0.64–1.32]	1.10 [0.70–1.82]	0.81 [0.60–1.12]	<0.01
LV ejection fraction, %	62 [60–66]	60 [57–65]	63 [60–67]	0.02
New York Heart Association class, n (%)				0.07
1	5 (2)	3 (3)	2 (1)	
2	171 (49)	42 (41)	129 (54)	
3	165 (48)	57 (56)	108 (44)	
4	2 (1)	-	2 (1)	
Intraoperative Characteristics				
Robot-assisted mitral valve surgery, n (%)	129 (38)	27 (27)	102 (42)	<0.01
Mitral valve replacement, n (%)	18 (5)	10 (10)	8 (3)	0.03
Left appendage closure, n (%)	28 (8)	7 (7)	21 (9)	0.67
Cardiopulmonary bypass time, min	140 [119–169]	152 [120–178]	137 [118–165]	0.03
Cross-clamp time, min	88 [77–104]	95 [78–115]	86 [77–100]	<0.01
Operative time, min	230 [205–270]	240 [200–280]	229 [205–265]	0.14
IABP, n (%)	2 (1)	2 (2)	-	0.09

Data are expressed as median [interquartile range] and count (percentage). Legend: PPUC = prolonged postoperative use of catecholamines; BMI = body mass index; COPD = chronic obstructive pulmonary disease; RDW = red blood cell distribution width; CRP = C-reactive protein; LV = left ventricular; NYHA = New York Heart Association; IABP = intra-aortic balloon pump; ICU = intensive care unit; AKI = acute kidney injury; MV = mechanical ventilation.

**Table 2:** Univariate and multivariate logistic regression analyses of higher postoperative hemodynamic instability.

	UNIVARIATE		MULTIVARIATE	
	Unadjusted OR [CI 95%]	*p-*Value	Adjusted OR [CI 95%]	*p-*Value
Red cell distribution width				
<13.2%	-	-	-	-
13.2–13.7%	1.40 [0.68–2.88]	0.36	1.26 [0.58–2.73]	0.56
13.8–14.4%	1.40 [0.68–2.85]	0.36	1.24 [0.57–2.68]	0.59
>14.4%	3.11 [1.58–6.11]	<0.01	2.62 [1.06–4.84]	0.03
EuroSCORE II	1.67 [1.28–2.19]	<0.01	1.38 [1.03–1.85]	0.03
Cross-clamp time	1.01 [1.01–1.02]	<0.01	1.01 [1.01–1.02]	<0.01
Preoperative atrial fibrillation	1.77 [1.04–3.01]	0.04	1.01 [0.54–1.87]	0.99
Robot-assisted mitral valve surgery	0.49 [0.30–0.82]	<0.01	0.53 [0.30–0.93]	0.03
Arterial hypertension	1.84 [1.14–3.00]	0.01	1.33 [0.77–2.29]	0.30
Mitral valve replacement	3.17 [1.21–8.29]	0.02	1.29 [0.43–3.86]	0.65
Preoperative ejection fraction	0.96 [0.92–0.99]	0.02	0.98 [0.94–1.02]	0.37

Hosmer and Lemeshow goodness-of-fit: *p* = 0.97.

## Data Availability

Data are available on request to the corresponding author.

## Supplementary Materials

The following supporting information can be downloaded at: https://www.mdpi.com/article/10.3390/jcm13195736/s1. Table S1. Postoperative characteristics of study population, according to hemodynamic instability. Table S2. Characteristics of catecholamine use, according to hemodynamic instability. Table S3. Characteristics of the study population without major bleeding, according to development of postoperative hemodynamic instability. Table S4. Univariate and multivariate logistic regression analysis of higher postoperative hemodynamic instability in subgroup of patients without major bleeding. Figure S1: Flowchart of the study.
