# Supplementary material for: Relationship between Preoperative Red Cell Distribution Width and Prolonged Postoperative Use of Catecholamines in Minimally Invasive Mitral Valve Surgery Patients: A Retrospective Cohort Study
Source: J Clin Med. 2024 Sep 26;13(19):5736. doi: 10.3390/jcm13195736 (PMC11476661; doi:10.3390/jcm13195736)
Supplement: Supplementary file 1 [file jcm-13-05736-s001.zip › jcm-3197973-supplementary.pdf]

Supplemental Material

Relationship between preoperative Red Cell Distribution Width and prolonged postoperative use of catecholamines in minimally invasive mitral valve surgery patients: A retrospective cohort study

Table S1. Postoperative characteristics of study population, according to hemodynamic instability

	**PROLUNGED POSTOPERATIVE USE OF CATECHOLAMINES (n=102)**	**NOT PROLUNGED POSTOPERATIVE USE OF CATECHOLAMINES (n=241)**	***p values***
Highest Lactate at 24 hours, mmol/L	4.0 [2.4-6.3]	2.6 [1.9-3.8]	<0.01
Re-exploration for bleeding, n (%)	9 (9)	3 (1)	0.01
Blood loss first 24 hours, ml	405 [260-670]	330 [250-455]	<0.01
ICU Stay, hours	47 [44-72]	43 [34-46]	<0.01
MV time, hours	8 [4-13]	4 [3-6]	<0.01
Postoperative AKI	16 (16)	6 (3)	<0.01
Postoperative length of stay, days	9 [8-12]	8 [7-10]	<0.01
Discharge at home, n (%)	52 (51)	169 (70)	<0.01
Hospital Mortality, n (%)	-	-	-

Data are expressed as median [interquartile range] and count (percentage). Legend: ICU = Intensive Care Unit; AKI = Acute kidney injury; MV = Mechanical Ventilation.

Table S2. Characteristics of catecholamines use, according to hemodynamic instability.

	**PROLUNGED POSTOPERATIVE USE OF CATECHOLAMINES (n=102)**	**NOT PROLUNGED POSTOPERATIVE USE OF CATECHOLAMINES (n=241)**	***p values***
Dobutamine, n (%)	22 (22)	6 (3)	<0.01
Dobutamine highest dose,	3.0 [3.0-4.0]	2.0 [2.0-2.5]	<0.01
Dobutamine hours of infusion	18 [15-22]	3 [3-4]	<0.01
Epinephrine, n (%)	76 (75)	38 (16)	<0.01
Epinephrine highest dose,	0.04 [0.03-0.05]	0.03 [0.03-0.05]	0.27
Epinephrine hours of infusion	16 [12-18]	6 [4-9]	<0.01
Norepinephrine, n (%)	44 (44)	20 (8)	<0.01
Norepinephrine highest dose,	0.05 [0.05-0.10]	0.05 [0.05-0.08]	0.58
Norepinephrine hours of infusion	14 [12-22]	5 [4-7]	<0.01

Data are expressed as median [interquartile range] and count (percentage).

Table S3. Characteristics of the study population without major bleeding, according to development of postoperative hemodynamic instability

	**PROLUNGED POSTOPERATIVE USE OF CATECHOLAMINES (n=60)**	**PROLUNGED POSTOPERATIVE USE OF CATECHOLAMINES (n=190)**	***p values***
Age, years	62 [55-71]	62 [51-70]	0.20
Female gender, n (%)	24 (40)	72 (38)	0.76
BMI	24.8 [22.6-26.7]	24.7 [22.4-26.8]	0.65
**Preoperative Characteristics**			
Atrial Fibrillation, n (%)	22 (37)	37 (20)	<0.01
Mitral valve stenosis, n (%)	2 (3)	5 (3)	0.68
Arterial Hypertension, n (%)	43 (72)	96 (51)	<0.01
Chronic Arteriopathy, n (%)	1 (2)	2 (1)	0.57
Diabetes, n (%)	3 (5)	10 (5)	1.00
Neurologic Disease, n (%)	4 (7)	18 (10)	0.61
Asthma/COPD, n (%)	4 (7)	13 (7)	1.00
Active Smoker, n (%)	9 (15)	24 (13)	0.66
Preoperative Hemoglobin	14.0 [12.8-14.9]	14.0 [13.0-14.9]	0.58
Preoperative Creatinine	0.91 [0.80-1.11]	0.90 [0.80-1.01]	0.28
Preoperative RDW	13.9 [13.5-14.8]	13.6 [13.1-14.1]	0.01
Preoperative RDW			0.01
<13.2%	9 (15)	52 (27)	
13.2-13.7%	13 (22)	48 (25)	
13.8-14.4%	14 (23)	53 (28)	
>14.4%	24 (40)	37 (20)	
Preoperative CRP	0.19 [0.06-0.32]	0.12 [0.07-0.26]	0.18
EuroSCORE II, score	0.97 [0.70-1.57]	0.82 [0.60-1.11]	0.02
Ejection Fraction, %	60 [58-65]	63 [60-67]	0.06
New York Heart Association class, n (%)			0.20
1			
2	2 (3)	2 (1)	
3	23 (38)	98 (52)	
4	35 (58)	89 (47)	

	-	1 (1)	
**Intraoperative Characteristics**			
Robotic assisted mitral valve surgery, n (%)	12 (20)	75 (40)	<0.01
Mitral Valve Replacement, n (%)	4 (7)	7 (4)	0.47
Left Appendage closure, n (%)	5 (9)	16 (8)	1.00
Cardiopulmonary bypass time, min	144 [123-175]	133 [113-159]	0.03
Cross-clamp time, min	97 [80-114]	85 [75-98]	<0.01
Operative time, min	230 [203-279]	223 [200-263]	0.19
IABP, n (%)	1 (2)	-	0.24
**Postoperative Characteristics**			
Highest Lactate at 24 hours, mmol/L	4.6 [2.8-7.4]	2.6 [1.8-3.6]	<0.01
Re-exploration for bleeding, n (%)	-	-	-
Blood loss first 24 hours, ml	300 [240-400]	295 [220-370]	0.59
ICU Stay, hours	46 [43-70]	43 [26-45]	<0.01
MV time, hours	6 [4-10]	4 [3-6]	<0.01
Postoperative AKI	9 (15)	2 (1)	<0.01
Postoperative length of stay, days	9 [8-11]	8 [6-10]	<0.01
Discharge at home, n (%)	30 (50)	132 (70)	<0.01
Hospital Mortality, n (%)	-	-	-

Data are expressed as median [interquartile range] and count (percentage). Legend: BMI = Body Mass Index; COPD = Chronic Obstructive Pulmonary Disease; RDW= Red blood Cells Distribution Width; CRP= C-Reactive Protein; LV = Left Ventricular; NYHA = New York Heart Association; IABP = Intraaortic balloon pump; ICU = Intensive Care Unit; AKI = Acute kidney injury; MV = Mechanical Ventilation.

Table S4. Univariate and multivariate logistic regression analysis to higher postoperative hemodynamic instability in subgroup of not major bleeding patients

	**UNIVARIATE**		**MULTIVARIATE**	
	**Unadjusted OR [CI 95%]**	***p values***	**Adjusted OR [CI 95%]**	***p values***
Red Cells Distribution Width				
<13.2%				
13.2-13.7%	-	-	-	-
13.8-14.4%	1.57 [0.61 – 3.99]	0.35	0.99 [0.36 – 2.71]	0.98
>14.4%	1.53 [0.61 – 3.83]	0.37	1.02 [0.38 – 2.72]	0.98
	3.75 [1.56 – 8.99]	<0.01	2.19 [0.85 – 5.65]	0.10
EuroSCORE II	1.73 [1.16 – 2.60]	<0.01	1.33 [0.85 – 2.08]	0.21
Cross-clamp time	1.02 [1.01 – 1.03]	<0.01	1.02 [1.01 – 1.03]	<0.01
Preoperative Atrial Fibrillation	2.39 [1.27 – 4.52]	<0.01	1.45 [0.71 – 3.06]	0.29
Robotic Assisted Mitral Valve Surgery	0.38 [0.19 – 0.77]	<0.01	0.45 [0.21 – 0.97]	0.04
Arterial Hypertension	2.48 [1.32 – 4.65]	<0.01	1.78 [0.88 – 3.60]	0.11

Hosmer and Lemeshow goodness-of-fit: p=0.20

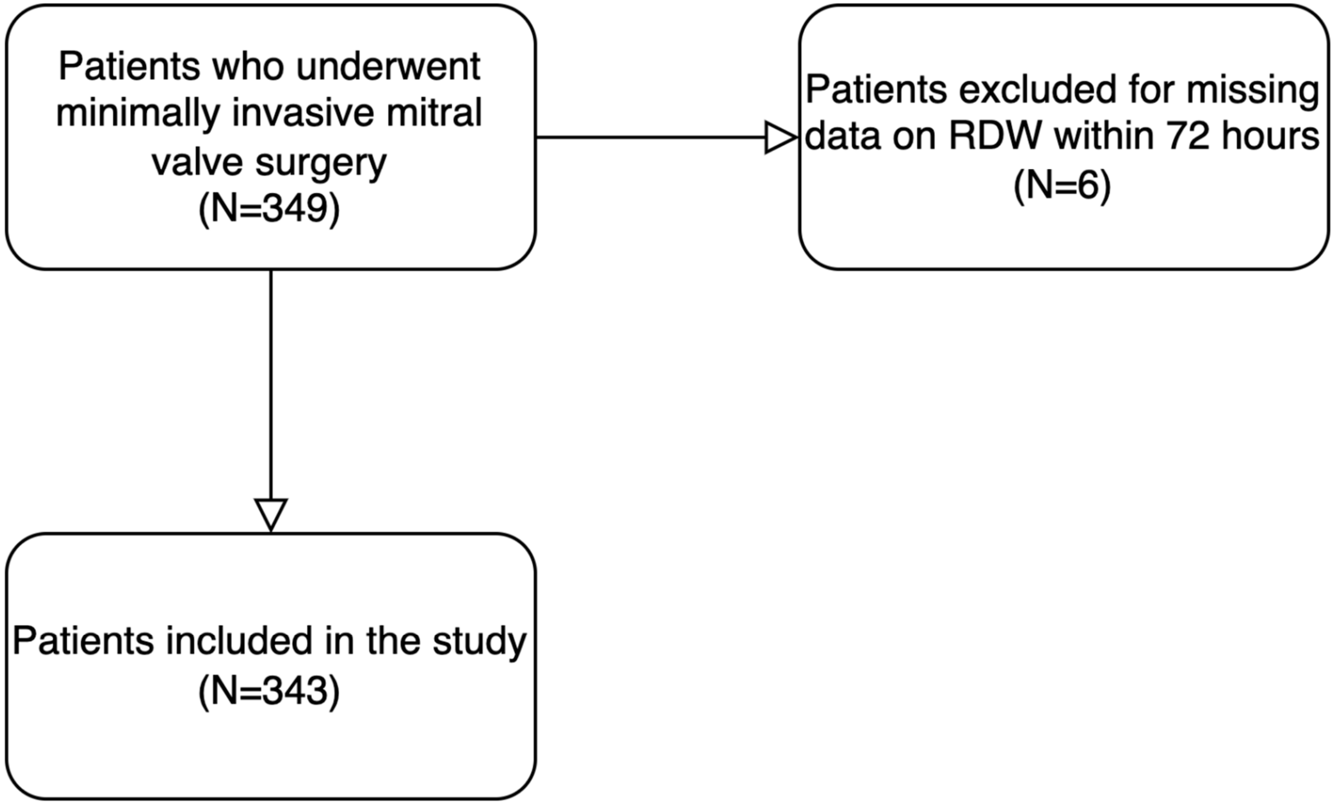

Figure S1. Flowchart of the study
